# Oxidative Stress and Antioxidants in Age-Related Macular Degeneration

**DOI:** 10.3390/antiox12071379

**Published:** 2023-07-03

**Authors:** Neetu Kushwah, Kiran Bora, Meenakshi Maurya, Madeline C. Pavlovich, Jing Chen

**Affiliations:** Department of Ophthalmology, Boston Children’s Hospital, Harvard Medical School, 300 Longwood Avenue, Boston, MA 02115, USA

**Keywords:** antioxidant, age-related macular degeneration, choroidal neovascularization, oxidative stress, reactive oxygen species, retinal pigment epithelium

## Abstract

Oxidative stress plays a crucial role in aging-related eye diseases, including age-related macular degeneration (AMD), cataracts, and glaucoma. With age, antioxidant reparative capacity decreases, and excess levels of reactive oxygen species produce oxidative damage in many ocular cell types underling age-related pathologies. In AMD, loss of central vision in the elderly is caused primarily by retinal pigment epithelium (RPE) dysfunction and degeneration and/or choroidal neovascularization that trigger malfunction and loss of photo-sensing photoreceptor cells. Along with various genetic and environmental factors that contribute to AMD, aging and age-related oxidative damage have critical involvement in AMD pathogenesis. To this end, dietary intake of antioxidants is a proven way to scavenge free radicals and to prevent or slow AMD progression. This review focuses on AMD and highlights the pathogenic role of oxidative stress in AMD from both clinical and experimental studies. The beneficial roles of antioxidants and dietary micronutrients in AMD are also summarized.

## 1. Introduction

Reactive oxygen species (ROS) are chemicals containing one or more unpaired electrons in their outer shell, such as superoxide, peroxide, and hydroxyl radicals as a few examples. As highly reactive free radicals, ROS are generated as byproducts of mitochondrial metabolism and by specialized enzymes [1]. During normal physiology, ROS serve as signaling molecules and are scavenged by antioxidants and antioxidative enzymes that inhibit oxidation [2]. In diseases, however, an imbalance between the production of ROS and the detoxifying antioxidant defense leads to oxidative stress, causing various deleterious effects at molecular, cellular and tissue levels, particularly during aging when the antioxidative enzyme defense capacity is declined.

Oxidative stress plays a significant role in the development of age-related eye disorders such as age-related macular degeneration (AMD), glaucoma and cataracts. While aging itself is one of the key risk factors in the development of these age-related eye diseases [3], and these diseases are clearly multi-factorial, excessive oxidative stress is detrimental in causing cellular damage in various ocular cell types that contribute to the disease onset. Antioxidants play an important role in cellular defense against oxidative stress and are beneficial in preventing disease progression and retaining vision in various age-related eye disorders, particularly AMD [4]. Hence, use of micronutrients with high antioxidant capacity can be promising preventive and therapeutic strategies in eye diseases associated with aging.

In this review, we summarize the role of oxidative stress and antioxidants in age-related eye diseases with a specific focus on AMD. An overview of both clinical and experimental research on AMD is provided regarding the pathogenic role of oxidative stress in both dry and wet AMD with particular emphasis on the most recent research advances in the past few decades. Evidence of antioxidants as feasible therapeutic approaches is discussed.

## 2. Production of ROS, Redox Homeostasis, and Their Impact on Aging

### 2.1. Imbalance of Redox Homeostasis Causes Oxidative Damage

**Figure 1:**
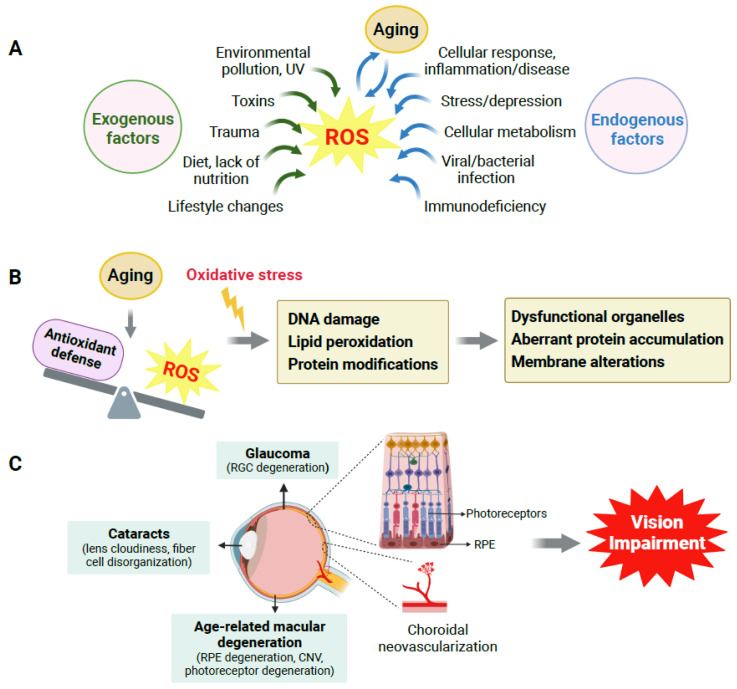
Aging, oxidative stress and their impact on age-related eye diseases. (**A**) Multiple exogenous and endogenous factors contribute to reactive oxygen species (ROS) generation, and aging plays a crucial role. (**B**) Oxidative stress is caused by an imbalance between ROS generation and the antioxidant defense system during aging. Oxidative stress (indicated by yellow lightning symbol) induces oxidative DNA damage, lipid peroxidation, and modifications in protein structure and functioning, which lead to dysfunctional organelles, aberrant protein accumulation, cell membrane alterations, and eventually cellular and tissue dysfunction. (**C**) In the eye, aging and associated oxidative stress cause oxidative damage to multiple types of ocular cells, which contribute to the onset and progression of age-related eye disorders such as age-related macular degeneration (AMD), glaucoma, and cataracts, all of which can ultimately cause vision loss in the elderly. CNV: choroidal neovascularization; RGC: retinal ganglion cell; RPE: retinal pigment epithelium. Created with BioRender.com.

Many exogenous and endogenous factors are sources of ROS production (Figure 1A). In humans, oxidative stress is highly influenced by exogenous factors, including lifestyle choices such as lack of physical activity, unhealthy diet, and nutritional deficiency. Environmental pollution, including exposure to UV, chemicals, and pesticides, also contributes to ROS production and excess oxidative stress. Endogenous factors such as cellular metabolism, genetic alterations, immunodeficiency, infection, inflammatory diseases, and aging itself also impact the production of ROS.

Generally, healthy cells have the endogenous antioxidative capacity of self-protection against ROS with antioxidant defensive enzymes such as superoxide dismutase (SOD), catalase, and glutathione peroxidase [5] that neutralize and reduce oxidation of free radicals. Dietary intake of antioxidant nutrients, such as vitamins C and E, and endogenous synthesis of antioxidant molecules, including glutathione, CoQ10, and ubiquinol, also help keep ROS under check [6]. However, excessive production of ROS and/or impaired antioxidant protection during diseases or aging may cause an imbalance in redox homeostasis, and the resultant oxidative stress can then lead to molecular and cellular oxidative damage.

Oxidative stress may alter and damage biomolecules including nucleic acids, proteins, lipids, and carbohydrates [7,8]. At the molecular level, damage to proteins may trigger structural modifications, thereby dampening protein function or catalytic activity of enzymes and impairing normal cellular function and metabolic pathways. Alterations in lipids, such as lipid peroxidation, also occur with oxidative damage. For example, lipid peroxidation products such as 4-hydroxynonenal (4-HNE) can form protein adducts that further precipitate oxidative protein damage and induce cell apoptosis. Malonaldehyde, another toxic product of lipid peroxidation, reacts with DNA and can cause genetic mutations. Mutations may also result directly from ROS-induced DNA base oxidation, which induces DNA double-strand breaks that further alter the DNA damage response and mutagenesis [8]. Together, these changes can negatively affect functions of cellular organelles and disrupt essential cellular processes [9] (Figure 1B).

At the cellular signaling level, ROS may activate many signaling molecules that negatively affect cell survival and growth and may eventually lead to cell death [10]. Oxidative damage by ROS activates diverse intracellular signaling cascades by modulating multiple transcriptions factors and enzymes such as nuclear factor kappa B (NF-κB), activator protein 1 (AP-1), cAMP response element-binding (CREB), and early growth response (EGR) protein [11]. These transcription factors travel from the cytoplasm to the nucleus within the cell and can bind with promoter regions of a particular target gene. This can significantly alter target gene expression and hence disrupt normal cellular homeostasis [12]. Furthermore, ROS may lead to damage in iron metalloproteins, which may prevent cell growth, survival, and proliferation [13].

### 2.2. ROS Accumulation Is One of Many Causes in Aging and Age-Related Disorders

Elevation in ROS levels is associated with the progression of aging [9,14], as the free radical theory of aging points toward the generation and lethal accumulation of ROS in cell components and tissues as a possible cause of aging-induced changes and age-related degenerative disorders [15]. Although there are limitations to this theory, broader cumulative cellular damage has also been proposed as a causal factor of aging [16]. Aging with associated gradual functional decline reflects cellular senescence, chronic inflammation, and the steady demise of normal cellular homeostasis [17,18,19]. Cellular senescence has been associated with ROS generation, which further mediates the crosstalk between ROS, inflammation and DNA damage signaling [20]. In addition, the proteosome plays a crucial role in the degradation of damaged proteins and their aggregates [21]. As damaged proteins accumulate during aging, they are cleared from the system via proteosome machinery. When this proteosome machinery becomes dysfunctional in aging, it also results in oxidative stress [21,22,23]. Moreover, during aging, the endogenous antioxidative reparative system may also become compromised or impaired, which further contributes to imbalances in redox homeostasis and thereby to oxidative stress.

Oxidative stress is an important contributing factor in the pathogenesis and progression of a variety of age-related disorders including neurodegenerative disorders in the brain and retinal degenerative disorders in the eye [9,24,25]. Aging and oxidative stress are contributing factors for the degeneration of neurons and may hence result in age-related neuronal and retinal degenerative diseases, which are often also associated with chronic neuroinflammation and microglial activation [26].

## 3. Age-Related Eye Disorders

### 3.1. Overview of Age-Related Eye Disorders

Several age-related eye disorders, namely AMD, glaucoma, and cataracts, commonly result in vision loss in the elderly. Each eye disease has distinctly different ocular cell types being impacted through aging, with damage to retinal ganglion cells (RGCs) in glaucoma, damage to the lens cells in cataracts, and in dry and wet AMD, damage to RPE and choroid respectively, which eventually leads to loss of the photoreceptors in AMD (Figure 1C). Oxidative stress is a common factor implicated in the degeneration of all these affected ocular cells and their disease pathophysiology.

AMD is a leading cause of vision loss in the elderly worldwide, and its prevalence increases with age [27]. As a chronic deteriorating disease, AMD manifests with gradual degeneration of the macula in the central retina to impair fine color vision. AMD is a leading cause of impaired vision in the United States, impacting over 14 percent of Americans aged 80 and older [27,28]. By one estimate, approximately 200 million individuals worldwide are affected by AMD [29]. While the risk factors of AMD are multifactorial with both genetic and environmental components, age-induced oxidative stress and cellular damage are major causes of RPE dysfunction in early AMD as well as of eventual RPE degeneration and choroidal neovascularization in late AMD. As a monolayer of supporting cells underneath the neurosensory retina [3], RPE provides vital trophic support to photoreceptors. Dysfunction and loss of RPE may cause photoreceptor loss in nearby regions and lead to vision loss.

In glaucoma, aging and oxidative stress are the leading causes of damaged and dysfunctional RGCs, the primary cellular site of glaucoma pathology. Axons of RGCs form the optic nerve, which is responsible for transmitting light signals from the retina to the brain. Glaucoma is age-related and causes gradual yet irreversible blindness in peripheral vision due to axon damage and death of RGCs via apoptosis [30,31], and it is often related to elevated ocular pressure. Glaucoma causes impaired vision in about 3.6 million individuals aged 50 years and above globally [32]. Glaucoma was found to be associated with elevated levels of ROS in affected eyes and with depletion of essential neurotrophic factors that trigger chronic neuroinflammation and RGC loss [33,34]. The antioxidant and anti-inflammatory nature of certain dietary foods and nutritional supplements such as vitamins, carotenoids, omega-3 fatty acids, flavonoids, and curcumin have been reported to show promising beneficial effects in experimental studies, yet clinical studies have been inconclusive, suggesting the need for additional investigation [35,36,37]. In addition, glutathione and flavonoids were reported to have significant protective effects against glaucoma [38]; however, more studies are needed to provide stronger evidence for clinical relevance.

Roughly 100 million eyes are expected to be affected with cataract globally [39]. Cataracts are any type of partial or entire opacification of the lens, which blocks light path and thereby impairs vision [40]. Any region of the lens such as the cortex, nucleus or posterior pole can be affected by age-related cataract formation, where nuclear and cortical cataracts are predominantly caused by oxidative stress from lifelong radiation exposure and other sources of ROS [41]. Aging is associated with increased protein aggregation in the lens, and oxidation is characteristic of age-related nuclear cataract [42]. Cataracts are caused by crystallin protein aggregation and disorganization in the lens nucleus fiber cells, which lead to loss of cell transparency and hence cataract formation [41]. While cataract surgeries are generally successful, antioxidants were also proposed as potential therapies to delay the onset and progression of cataract formation, although many human and experimental studies have produced mixed and inconsistent results [43] regarding the benefits of natural compounds for the treatment of cataract formation, including vitamins C and E [44,45,46]. Additional studies are warranted.

### 3.2. Effects of Aging on the Eye, Retina and RPE

Many organs become dysfunctional during aging, and the eye is no exception. In the eye, the detrimental effects of aging are not limited to the essential components of the retina and the lens, but almost all other ocular structures including the cornea and ocular surface are also affected during aging [47]. Aging leads to the accumulation of free radicals, which further cause oxidative damage and decline of physiological functions in many cell types in the eye, supporting the oxidative stress theory of aging [48].

In various hereditary and age-related retinal diseases, neuronal loss results in functional decline in visual acuity. For instance, the death of photoreceptors in retinitis pigmentosa [49], loss of retinal ganglion cells (RGCs) and their axons in glaucoma [50], age-dependent decreases in the density of rod bipolar cells [51], and RPE degeneration and consequent photoreceptor loss in AMD [52] are all examples of neuronal loss leading to functional detriments in vision.

**Figure 2:**
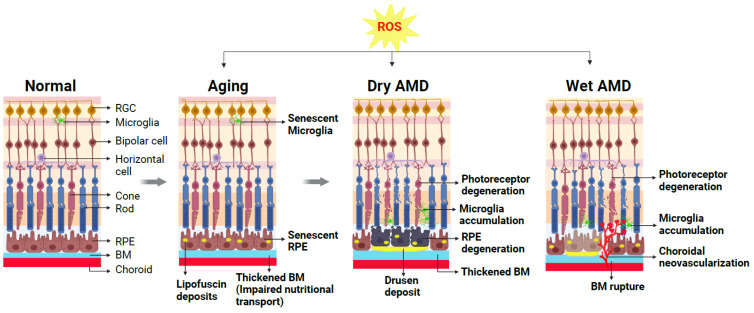
Schematic representation demonstrating cellular changes in normal and aging retinas and retinas with dry AMD or wet AMD. A normal healthy retina is composed of multiple layers of retinal neurons including photoreceptors (rods and cones), bipolar cells, horizontal cells, retinal ganglion cells (RGCs), and supporting cells such as microglia, retinal pigment epithelium (RPE) cells, and choroid beneath the RPE. During normal aging, excessive reactive oxygen species (ROS) and resultant oxidative damage contribute to age-related changes in the retina (gray arrows indicate progression of retinal changes due to aging/ROS/AMD disease). Normal functions of RPE and microglia are disturbed, and these cells become dysfunctional. The accumulation of lipofuscin deposits in the RPE and impaired nutritional transport due to thickened BM are hallmarks of the aging retina, setting the stage for AMD onset. The presence of large lipid-enriched sub-RPE deposits known as drusen is a diagnostic marker for AMD. Advanced dry AMD is characterized by degenerating RPE and nearby photoreceptor degeneration. Altered choroid structure and increased microglia accumulation in the subretinal spaces indicative of chronic inflammation are also evident. In the wet form of AMD, pathological growth of abnormal choroidal blood vessels occurs through ruptured BM into the sub-RPE and subretinal space, causing exudates, hemorrhage, and thereby degeneration of the nearby photoreceptors. Created with BioRender.com.

In RPE, aging leads to senescent RPE with altered mitochondrial metabolism [53]. Additionally, aging causes a decrease in the density of RPE cells [54] and buildup of harmful intra- and extracellular proteins and lipid deposits called lipofuscin, which consists of residues of lysosomal digestion [55]. Excessive production of melanin in RPE and accumulation of aggregates such as lipofuscin and melanosomes may further contribute to various retinal diseases such as AMD, Stargardt disease, retinitis pigmentosa, and Best’s disease [27,56]. In additional to RPE cell dysfunction, the thickening of Bruch’s membrane (BM), due to the accumulation of proteins and lipid aggregates, is also a hallmark of aging retina [57]. Age-related changes observed in the histology of human donor eyes include RPE hyperpigmentation, thickening of BM, and disorganized RPE and photoreceptor layers [58]. With increasing age, the lining of the choroid also becomes thinner, with a reduction in both choroid capillary density and consequently decreased blood flow [59]. This choroidal change may impact RPE and photoreceptor health. Furthermore, the number of photoreceptors including both rods and cones also decreases with age (Figure 2) [60,61,62,63].

At the molecular level, studies exploring age-related gene expression in young versus old human donor retinas have identified the differential expression of genes related to cell survival, growth, and proliferation, with age-related decline in genes regarding retinal protection and survival, such as brain derived neurotrophic factor (*BDNF),* X-linked inhibitor of apoptosis (*XAF1*), cadherin (*CDH8*), and protein tyrosine kinase (*PTK2*) [64]. On the other hand, aging leads to the upregulation of genes associated with cell death and apoptosis, such as chloride intracellular channel 4 (*CLIC4*) and nuclear receptor co-repressor 2 (*NCOR2*) [64]. These age-dependent molecular changes may set the stage for the development of age-related retinal diseases such as AMD and glaucoma.

Numerous epidemiological studies have demonstrated that aging itself, beyond other genetic and environmental factors, is a major risk factor for many types of eye disorders, including AMD, glaucoma, and cataract formation. With AMD being one of the most common causes of visual impairment, AMD will be the focus of the remaining parts of this review.

## 4. Age-Related Macular Degeneration: Classification, Pathophysiology, and Current Treatment

### 4.1. AMD: Clinical Classification

The clinical characterization of AMD stages is based on the size and density of drusen and pigmentary status. Drusen consist of the extracellular accumulation of lipid-enriched substance, which deposit between the BM and RPE cells. These drusen serve as a hallmark for early (>63 µm diameter) or intermediate (>125 µm diameter) stages of the disease [65]. The advanced or late stage of AMD has two forms (dry and wet) and is characterized by the presence of RPE atrophy, known as geographic atrophy (GA) in atrophic (or dry) AMD, or choroidal neovascularization (CNV) in neovascular (or wet) AMD in combination with drusen accumulation [66].

### 4.2. AMD: Dry and Wet Forms

Advanced AMD has two types, dry AMD, and wet AMD. Dry AMD is the most common form of AMD, which accounts for approximately 80–90% of all AMD cases. RPE degeneration is the hallmark of late-stage dry AMD progression. Dry AMD is characterized by the presence of small yellow drusen deposits in the subretinal space that progressively increase in density and size in combination with geographic atrophy in advanced dry AMD. RPE cells are located between the neural retina and the choroid and participate in the transport of nutrients and metabolites as well as the removal of metabolic waste to support photoreceptors. RPE is additionally responsible for the recycling of visual pigments and renewing the photoreceptor outer segment via phagocytosis [67]. This normal function of RPE and substance exchange are increasingly impaired as BM is thickened with lipid deposition during aging. In AMD retinas, RPE cells lose their pigmentation and initially function normally, yet they gradually become dysfunction and die, causing geographic atrophy with associated photoreceptor and choriocapillaris degeneration and further vision loss [52,68] (Figure 2).

In contrast to dry AMD, the less common neovascular or wet form of AMD is characterized by CNV and CNV-associated loss of photoreceptors and RPE cells. CNV is the abnormal extension of blood vessels from the choroid intruding through BM into the sub-RPE or sub-retinal space [69], induced by abnormally high levels of vascular endothelial growth factor (VEGF) [70]. Increased production of VEGF results in the pathological growth of choroidal blood vessels with associated hemorrhage and exudates and thus results in rapid and profound loss of central vision [71] (Figure 2). Three CNV subtypes in wet AMD have been identified according to the origin and location of CNV in the retina. Type 1 CNV occurs beneath the RPE cells, whereas type 2 arises when CNV infiltrates through RPE into the subretinal space toward the photoreceptor layer [72]. Retinal circulation is impacted in the third type of CNV, identified as retinal angiomatous proliferation (RAP), which initiates as retinal neovascularization and progresses into the subretinal space and forms an eventual anastomosis among the choroidal and retinal vessels [73].

### 4.3. AMD Pathogenesis: Genetic and Environmental Factors

AMD is an age-driven multifactorial disease that exhibits susceptibility toward genetic components [74]. Most genes associated with AMD development and susceptibility are associated with innate immunity and inflammation pathways, particularly the complement system and complement factor H (*CFH*) [75]. Apart from *CFH*, other components of the complement system such as complement 3 (*C3*, complement C2 (*C2*)/complement factor B (*CFB*) [76,77] and complement factor H-related 1, 3 (*CFHR1/CFHR3*) [78] also exhibit significant association with AMD development and progression. Another major genetic variant associated with AMD is *HRTA1* (high-temperature requirement A serine peptidase 1)/*ARMS2* (age-related maculopathy susceptibility 2). *ARMS2* and *HTRA1* genes are both located closely on chromosome 10q26, and individuals with genetic variations associated with these genes are among the most susceptible to AMD [79]. *HTRA1* encodes a heat shock serine protease and may regulate angiogenesis by modulating transforming growth factor-β (TGF-β) [80]. On the other hand, *ARMS2* has been implicated in regulating retinal mitochondrial function [81] and complement activation [82].

Development and progression of AMD are also strongly influenced by environmental factors such as smoking [83], high-fat diet [84], alcohol consumption [85] and obesity [86]. Many of these environmental factors including sunlight exposure, poor diet, and smoking are associated with the induction of oxidative stress and thereby may contribute to AMD pathology [87,88,89]. In addition, the lipid-laden drusen are highly vulnerable to oxidation by reactive oxygen species, thereby triggering oxidative damage. These factors have robust effects on oxidative stress and subsequent inflammation. The existing literature strongly supports an association between oxidative stress and inflammation, both of which play compelling roles in etiology of AMD [90], where oxidative stress may activate an innate immunity complex in the eye. For instance, in lipid-rich drusen, malondialdehyde (MDA), a common lipid peroxidation product, may form a complex with CFH to influence the complement pathway [91,92].

### 4.4. Current Available Treatment for AMD

Advancement in intravitreal anti-VEGF therapies in the past few decades has revolutionized the treatment of the wet or neovascular forms of AMD [93]. Increases in VEGF and other proangiogenic factors are a primary cause of CNV formation, the blinding feature in neovascular AMD [94]. Several anti-VEGF drugs are being used for the treatment of neovascular AMD [95] as the current standard of care, which have displaced prior treatment options including photodynamic therapy and laser photocoagulation. The first anti-VEGF agent approved for the treatment of wet AMD was pegaptanib, which inhibits just one VEGF isoform (VEGFA165). Later, other VEGF blockers, such as ranibizumab and bevacizumab, which inhibit entire isoforms of VEGFA, have replaced pegaptanib with significantly better visual results [96,97,98]. The current best available treatment for wet AMD is intravitreal injections of anti-VEGF therapies of aflibercept and ranibizumab [99]. Overall, these anti-VEGF agents are well accepted and effective in treating neovascular AMD, with reduction in CNV formation and improved visual ability [100]. However, limitations exist, including the need for frequent injections and treatment cost. Some patients have incomplete response or remain unresponsive to anti-VEGF treatment, and long-term safety and potential detrimental effects are still concerning [101,102]. Interactions of anti-VEGF treatment with other pathogenic pathways including inflammation and fibrosis also need additional investigation [102,103,104].

For dry AMD, on the other hand, the only current FDA-approved treatment is pegcetacoplan, an anti-complement therapy with targeted inhibition of C3, recently approved in 2023. Pegcetacoplan was shown to slow the progression of geographic atrophy by ~20% in clinical trials [105], yet visual function improvement has not yet been observed. This necessitates the need for the search of more potent and effective treatments to stop or slow the progression of geographic atrophy in dry AMD.

Overall, despite the effectiveness of anti-VEGF therapies and advances in anti-complement therapies, developing new or combinational therapies targeting other pathways to address the root causes of AMD are still much needed, including those involved in lipid dysregulation, immune and inflammatory dysfunction, and oxidative stress.

## 5. Presence of Oxidative Stress Markers in Clinical AMD

As oxidative stress is one of the major factors contributing to and worsening the pathological events occurring in AMD, many physiological conditions unique to the retina environment facilitate ROS generation and oxidative stress. These include high polyunsaturated fatty acid content in the photoreceptors for lipid peroxidation, high oxygen demand and mitochondrial metabolism, extensive light exposure to the retina, and the presence of lipofuscin [106].

In dry AMD characterized by RPE degeneration in late disease progression, the RPE provides protection to the retina against light-induced oxidative damage by absorbing excess photons. The RPE also has a high oxygen consumption rate from their high mitochondrial oxidative metabolism. RPE cells are hence highly susceptible to oxidative stress [107]. While RPE cells have their own antioxidative machinery to protect against oxidative stress, aging may impair RPE antioxidative machinery, making it less effective in combating oxidative damage [108,109]. Oxidative stress-induced RPE damage thus plays an important role in the progression of dry AMD [109].

Clinical studies in AMD patients with post-mortem donor eyes have provided compelling evidence supporting the presence of DNA, protein, and lipid biomarkers of oxidative stress in AMD. The presence of oxidative DNA damage including 8-hydroxy-2-deoxyguanosine (8-OHdG) was identified in AMD donor eyes, especially in dry AMD with RPE atrophy [110]. Since the photoreceptors’ outer segments are lipid enriched with docosahexaenoic acid (DHA) [111], enhanced levels of carboxyethylpyrrole (CEP) protein adducts, a biomarker of DHA-containing lipid peroxidation, in the BM of AMD donor eyes also indicate high vulnerabilities toward oxidative stress in AMD eyes compared to non-AMD [112,113,114]. In addition, increased CEP levels (~60% elevation) were detected in ELISA in dry AMD eyes compared to control donor eyes [115,116]. Yet no visible difference in total 4-hydroxy-2-nonenal (HNE) modified protein was found among different regions or during the four progressive stages of AMD in human donor eyes [117]. In another study, proteomic and genomic biomarker analysis in AMD donor eyes showed a 60% increase in plasma CEP levels, associated with elevated ARMS2, CFH, and complement C3 in dry AMD [118]. In addition to the identification of oxidative stress markers locally in the eyes, high levels of malondialdehyde (MDA), 8-OHdG and protein carbonyls were also found in the serum from AMD patients, suggesting systemic elevation of oxidative stress markers in AMD patients [119].

Beyond nuclear DNA oxidative damage, AMD donor eyes also showed RPE mitochondrial DNA damage due to oxidative stress [120,121], suggesting that an imbalance in RPE mitochondrial homeostasis and metabolic dysfunction due to oxidative stress may also lead to macular degeneration in AMD [122]. To this end, mitochondrial defects in RPE cells are being recognized as one of the major features in AMD pathology. This notion is supported by studies of mitochondrial and glycolytic functions in RPE, which were reduced in RPE cells isolated and cultured from AMD donor eyes compared with non-AMD eyes [123], with AMD-derived RPE cells showing lower levels of glutathione and ATP. Moreover, significantly higher levels of peroxisome proliferator-activated receptor-gamma coactivator 1α (PGC-1α) protein in these cells as compared to healthy RPE may also reflect susceptibility and compensatory response toward oxidative stress [123]. PGC-1α has beneficial and protective effects on mitochondrial metabolism and antioxidant capacity [124], which might enhance resistance to and protection against oxidative stress. Another compensatory mechanism of RPE cells to protect against oxidative stress is elevated levels of antioxidant enzymes that scavenge for ROS. Antioxidant enzymes such as catalase, copper- and zinc-containing SOD (CuZnSOD or SOD1), and manganese superoxide dismutase (MnSOD or SOD2) were found to be upregulated in immunoblots of AMD donor eyes in the early and intermediate stages of AMD, which may reflect a compensatory mechanism of upregulating pro-survival signaling to counter increased ROS production in AMD eyes [125]. Hence, an upregulation in antioxidative pathways might be a pro-survival mechanism in response to increased oxidative stress.

Investigations of wet AMD patients, on the other hand, have reported significantly increased levels of total oxidant status in the serum and decreased levels of total antioxidant status [126]. Similar studies also confirm a significant increase in total oxidant status levels in the serum of wet AMD patients [127], suggesting that increased oxidative stress and reduced antioxidant defense in wet AMD patients contribute to AMD progression. Levels of SOD, glutathione peroxidase (GPx) and glutathione reductase (GR) were lower in the serum of wet AMD patients of the age group 55–82 years compared with age-matched healthy control subjects [128], and SOD, catalase and GPx activities were also lower in erythrocytes of AMD patients vs. controls [129], indicating a strong correlation between wet AMD and oxidative stress and potentially dampened antioxidant enzyme functions in AMD. Yet other studies found upregulated levels of SOD1 in different populations, potentially reflecting a compensatory response [130,131]. Together, all these findings from clinical studies support the presence of oxidative biomarkers in AMD and a major role regarding oxidative stress in AMD pathology.

## 6. Experimental Studies of AMD Models Support the Pathogenic Role of Oxidative Stress in AMD

As AMD is a complex chronic disorder involving many genetic and environmental factors, the development of reliable animal models that can mimic all (early and late) pathological features of AMD has been challenging. Part of the difficulties are due to anatomical differences between commonly used research animals (e.g., rodents) and humans, as well as the chronic nature of the disease [132]. Nevertheless, many genetic and non-genetic models have been established that have facilitated the investigation of AMD pathogenesis, including the pathogenic role of oxidative stress.

### 6.1. Genetic Models of Dry AMD Reflect Oxidative Stress and Oxidative Damage

An accurate dry AMD model needs to recapitulate some if not all of the histological features observed in human AMD eyes, including drusen deposits, basal laminar deposits, thickening of BM, RPE structural and functional alteration and atrophy, accumulation of macrophages/microglia, accumulation of activated complement system proteins, and damage to photoreceptors [133,134]. Addition features include the accumulation of lipofuscin with high levels of N-retinylidene-N-retinylethanolamine (A2E), a fluorophore in RPE cells [135], and the functional decline of photoreceptor atrophy, which can be assessed by electroretinography (ERG) signals [136,137]. Based on these criteria, multiple genetic animal models were developed to study dry AMD, including targeted mutations in several essential factors in redox regulation.

Nuclear factor, erythroid-2-related factor 2 (NRF2), is a transcription factor with a crucial role in maintaining redox homeostasis by regulating antioxidant enzymes [138]. *Nrf2*^−/−^ mice develop several AMD-like features such as ROS accumulation, retinal dysfunction, photoreceptor degeneration, and inflammation [139]. Hence, defective NRF2 signaling is highly related to the vulnerability of RPE and photoreceptors to oxidative stress [140]. Superoxide dismutase (SOD) is a potent antioxidant enzyme expressed in three forms. SOD1 is cytosolic and is highly expressed in the retina, while mitochondrial SOD2 and extracellular SOD3 are expressed at relatively low levels in the retina [141]. These genes have been associated with an increased risk of AMD, which is in line with clinical studies [125,141]. *Sod1*^−/−^ and *Sod2* knockdown mice both showed increased drusen, RPE degeneration and disorganization of photoreceptors as compared to wildtype controls [142,143,144]. Nearly 10% of *Sod1*^−/−^ mice also exhibited evidence of CNV observable by fundus examination [142,143]. Additional models associated with oxidative damage include *ceruloplasmin/hephaestin*^−/−^ double knockout mice. Ceruloplasmin is a ferroxidase involved in facilitating iron export from cells, which is an effective initiator of oxidative stress, and *ceruloplasmin/hephaestin*^−/−^ double knockout mice exhibited iron overload with progressive retinal degeneration, modeling some AMD-like features [145,146].

Beyond direct regulators of oxidative stress, other complement-, lipid- and inflammation-associated animal models of AMD are also associated with oxidative stress. Several genetic models focusing on the complement and lipid pathways were developed due to their genetic association with human AMD. *Cfh*^−/−^ mice lacking CFH, a regulator of alternative pathways of the complement system, show AMD-like pathological features such as mitochondrial abnormality, visual function reduction in ERG amplitude, and increased subretinal deposits [147,148]. CFH was linked with AMD in part through interacting with oxidized phospholipids, hence modulating oxidative stress and inflammation [149]. In addition, AMD-risk-associated CFH variant Y402H was studied in transgenic mouse lines with the *ApoE* promotor for lipid dysregulation [150], and they develop progressions of AMD-like pathologies such as RPE abnormalities, basal laminar deposits and altered lipoprotein levels [151]. One lipid-related model for AMD pathologies is *ApoE*^−/−^ mice. ApoE is a major element of lipid-transporting particles. ApoE deficiency causes elevated levels of circulating cholesterol in *ApoE*^−/−^ mice [152,153]. Histological studies have demonstrated thickening of the BM and presence of membrane-bounded material resembling basal linear deposits in ApoE deficient mice [154]. Vulnerability to oxidative stress in *ApoE*^−/−^ mice is indicated by increased lipid peroxidation [155]. Moreover, targeted replacement mice expressing human ApoE isoforms have demonstrated that APOE2 exacerbates, and APOE4 protects against, AMD-like pathologies, including subretinal inflammation in an isoform-specific manner, consistent with their genetic disposition in human AMD [156]. Chemokines associated with microglia are also studied in AMD models for their link with inflammation. *Cx3cr1*^−/−^ mice, lacking CX3CR1, a chemokine in retinal microglia, exhibit subretinal accumulation of microglia and deposits in aged mice and induce retinal degeneration [157].

### 6.2. Non-Genetic Models of Chemically Induced Oxidative RPE Damage Represent Features of Dry AMD

In addition to genetic models, non-genetic models with chemically induced RPE damage are often investigated to probe the role of oxidative stress in RPE degeneration relevant to dry AMD. CEP, an adduct generated from the oxidation of omega-3 fatty acid DHA, was found in AMD donor eyes [112]. C57BL/6 mice immunized with CEP-modified mouse serum albumin (CEP-MSA) have lesions in RPE cells resembling geographic atrophy in dry AMD [158], suggesting a key role of oxidative damage in RPE degeneration. Injection of NaIO_3_, a strong inducer of acute oxidative stress, has been widely used as a model of RPE damage that produces selective toxic effects to RPE cells including RPE atrophy, secondary photoreceptor damage and retinal thinning in mice [159]. Intravenous injection of low levels of NaIO_3_ (20 mg/kg) in C57BL/6J mice has a transient effect on altered RPE cell morphology and ERG signals [160] and on abnormal visual behavior [161]. In this model, the severity of NaIO_3_-induced RPE damage is dose- (ranging from 25 to 100 mg/kg) and time-dependent. Hence, the pathological severity can be titrated based on the experimental needs [162]. Related models in rats and swine were also generated, which demonstrated similar NaIO_3_-induced RPE damage as observed in mice [163,164]. Cigarette smoking is a well-documented risk factor in AMD. Cigarette smoke extract promotes cellular senescence, proteolysis and autophagic impairment in RPE cells, all of which have been mechanism linked with AMD pathogenesis [165]. Chronic exposure of mice to cigarette smoke for 6 months causes RPE oxidative damage, ultrastructure changes and RPE apoptosis [166], suggesting detrimental effects of cigarette smoking in RPE oxidative stress and damage. Since electronic cigarettes (e-cigarettes) have been globally promoted and are currently widely used to replace conventional cigarettes, the effects of nicotine-free e-cigarette vapor has been evaluated in mice. These mice demonstrated inflammatory (IL-1β, TNFα, iNOS) and angiogenic (VEGF, PEDF) responses that were more prominent in RPE and in the choroid than in retinal tissue, reflecting a strong correlation with AMD [167]. Together, these non-genetic chemically induced models support a strong pathogenic role of oxidative damage in both RPE degeneration and CNV.

### 6.3. Recent Experimental Studies Have Established New Dry AMD Models with Identification of New Molecular Regulators of Oxidative Stress

The development of the oxidative stress-related dry AMD models has greatly enhanced our understanding of the role and mechanisms of oxidative damage in AMD pathogenesis. Investigations utilizing these model systems have probed various other aspects of oxidative stress in RPE damage, including necrosis. Animal models of oxidative stress as mentioned above have been shown to promote RPE dysfunction, apoptosis, and necrosis, associated with increased accumulation of drusen, complement pathway activation, local inflammation, and immune reactions [168].

More recent studies have identified new molecular players in regulating oxidative stress and damage in RPE. Investigation of proteasomal and autophagic machinery established *NRF-2/PGC-1α* double knockout mice as a model of dry AMD, showing elevated oxidative stress markers 4-HNE, endoplasmic reticulum (ER) stress, and damaged mitochondria [169]. Redox-sensitive transcription factor REV-ERBα protects RPE from age- and oxidative stress-induced RPE damage in mice via promoting *Nrf2* transcription and subsequent antioxidant response, where systemic and RPE-specific REV-ERBα-deficient mice demonstrated age-dependent RPE degeneration [170]. In a NaIO_3_-induced RPE damage model, *Rev-erbα*^−/−^ mice also exhibit more severe RPE atrophy, apoptosis, and photoreceptor damage than WT, whereas REV-ERBα activation protects against NaIO_3_-induced RPE damage [170]. In addition, in human AMD patients and in mouse models with dry AMD-like features, LCN2 (lipocalin 2) mediates the interconnection between the activation of inflammatory response and increased oxidative stress [171]. X-box binding protein 1 (XBP1) is a major transcription factor that regulates ER stress and antioxidant defense in RPE cells. RPE-specific XBP1 knockout mice showed reduced antioxidant enzymes and increased oxidative stress [172]. These developments have further enhanced our understanding of antioxidative defense regulatory pathways in RPE, with new insights into additional molecular targets for better approaches to counter oxidative stress in AMD.

### 6.4. Animal Models of Wet AMD: Roles of Oxidative Stress

Currently, available preclinical animal models to study CNV are limited. Several well-known models are dependent on the commonly used laser-induced CNV model, or the less common Matrigel-injection based CNV model. These models cause direct mechanical and acute injury to the RPE/BM complex or alter the RPE and its adjacent environment to induce CNV formation [132]. A few genetic models with spontaneous CNV formation have also been developed. All these models have facilitated the study of oxidative stress in CNV formation.

The mouse model of laser-induced CNV has been extensively used to study the neovascular aspect of AMD. In this model, laser beams damage the BM, resulting in the infiltration of choroidal vessels into the subretinal space at the site of injury [173]. Typically, four CNV lesions are produced in one eye with laser photocoagulation in mice aged 6–8 weeks [174]. Then, a 647 nm high-powered krypton laser is used to rupture the BM [175], and CNV lesions are evaluated 1–2 weeks post laser. In another study, a krypton laser was used to generate three lesion sites, where CNV formed in 87% of lesion sites at 2 weeks post laser [176]. Use of this model has laid the foundation for establishing the experimental basis for anti-VEGF therapy in current wet AMD treatment. Yet this model mimics only the neovascular features without considering the aging aspect of AMD.

Several studies have used subretinal injection of Matrigel or other substances to induce CNV [177]. The Matrigel mixture is in liquid form at 4 °C, and after subretinal injection in rabbits, it solidifies when the temperature rises to 37 °C [178]. In this solid form, Matrigel gradually releases angiogenic factors, which recruit choroidal vascular growth into subretinal space with associated vascular leakage. Additional AMD experimental models utilize the subretinal injection of components of CNV lesions such as macrophages to induce CNV [179,180,181]. Subretinal injection of lipid hydroperoxide has led to CNV formation in a rabbit model [182], and oxidized lipids such as (13(S)-hydroperoxy-9Z,11E-octadecadienoic acid (HpODE) in a rat model has led to photoreceptor and RPE degeneration [183,184].

In addition to the induced CNV models, spontaneous CNV formation was also found in several genetic models. Overexpressing VEGF in RPE cells was found to induce CNV [185], as RPE are known as a major cell type in secreting VEGF, while dysfunctional RPE or disrupted RPE polarity plays a crucial role in CNV formation and pathogenesis [186]. Other animal models of wet AMD or RAP comprise transgenic mice with the bovine rhodopsin promoter coupled with the human VEGF gene. These transgenic mice show growths of intraretinal and subretinal vessels originating from the retinal capillaries, which extend toward the photoreceptors into the subretinal space [187]. Mice deficient in very low-density lipoprotein receptor (VLDLR) have been recognized as a model for RAP and wet AMD regarding the development of subretinal neovascularization [188]. Loss of the *Vldlr* gene in mice increases retinal angiogenesis and abnormal neovascularization [189,190]. Similarly, the spontaneous mutant strain JR5558 also produces spontaneous CNV and RAP formation from retinal vessel origin, associated with RPE structural damage and dysfunction [191], and resembling the observations in VLDLR deficient mice. Albeit of unknown genetic causes, JR5558 strain represents a useful model to investigate the molecular and cellular processes associated with CNV and RAP [191]. In addition, subretinal administration of oxidized low-density lipoprotein (oxLDL) in C57/BL6 mice produces CNV-like lesions with increased VEGF levels and neovascularization [192].

Investigations on human patients and donor eyes have offered significant insight into AMD pathology, and studies on these different animal models of CNV are also crucial for preclinical evaluation [193]. An experimental study indicated that subretinal injection of human lipid hydroperoxide (HpODE) induced oxidative damage to RPE cells and retina in Sprague–Dawley (SD) rats and increased the production of VEGF leading to CNV formation [183]. This indicates a strong link of oxidative damage in RPE with CNV formation. In NRF2-deficient C57BL/SV129 mice, features of human AMD-like pathologies include severe RPE degeneration and evident CNV formation [194], suggesting an important role of NRF2-dependent antioxidant regulation in CNV formation. *C3aR*^−/−^*/C5aR*^−/−^ mice lacking both C3a and C5a receptors have also been utilized to investigate a crucial role of C3a and C5a and their receptors in advancing CNV formation [195,196], where these immunological factors also influence choroidal angiogenesis. A link between oxidative stress and *HTRA1* expression was also investigated to highlight the connection between oxidative stress and genetic disposition in AMD [192,197]. Oxidative stress induces *HTRA1* expression in the human RPE cell line ARPE-19 [198]. Additionally, the OxLDL model of CNV shows increased expression of *HTRAT1*, which generates oxidative stress, and inflammatory and angiogenic effects; thereby, the combination of oxLDL and *HTRA1* increases CNV lesion size [192]. 

**Figure 3:**
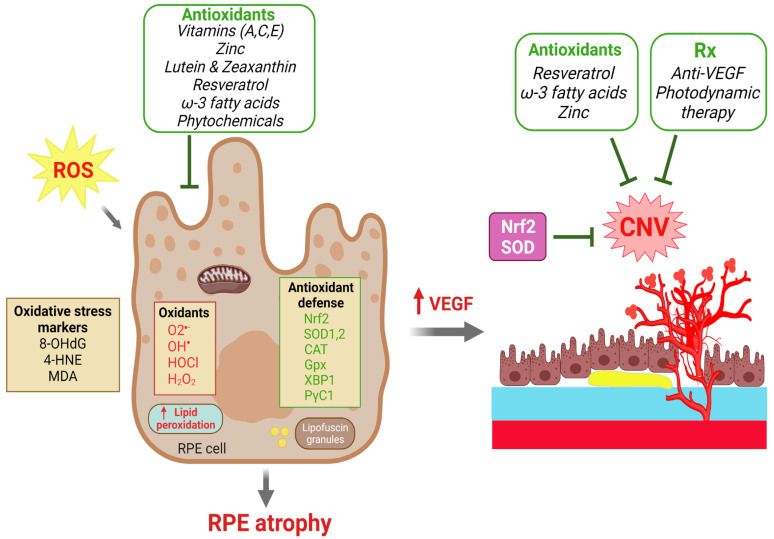
Roles of dietary antioxidants and antioxidant defense in protecting against RPE degeneration and choroidal neovascularization (CNV) in AMD. Excessive reactive oxygen species (ROS) and oxidants cause oxidative damage in the RPE as reflected by increases in oxidative stress markers and lipid peroxidation that eventually leads to RPE atrophy. Upward pointing red arrows indicate a resultant upregulation of the indicated biomolecule. RPE dysfunction and increased VEGF production further lead to CNV formation. The effects of ROS are countered intrinsically by antioxidant reparative defense systems with antioxidant enzymes to scavenger ROS. Multiple dietary antioxidant supplements have been investigated in both clinical trials and experimental studies that have demonstrated protective effects to prevent RPE atrophy and to slow the progression of AMD. CNV formation can be treated by anti-VEGF and photodynamic therapies. Therapeutic strategies against oxidative stress by targeting Nrf2, SOD, catalase and other antioxidants may have protective effects on both RPE degeneration and CNV. Created with BioRender.com.

The laser-induced CNV model has been utilized to investigate multiple oxidative factors in CNV formation. Solute carrier family 7 member 11 (*SLC7A11*), known as a major transporter of cystine/glutamate, plays a crucial role in regulating lipid peroxidation and preventing ferroptosis. In the laser-induced CNV model, *SLC7A11* reduces ferroptosis in RPE cells. Inhibition/knockdown of *SLC7A11* enhances the level of lipid peroxidation, decreases the viability of ARPE19 cells and increases laser-induced CNV size in C57BL/6 mice [199]. RS9, an activator of NRF2, reduced CNV lesions via the suppression of VEGF expression in a monkey model [200]. Malondialdehyde (MDA) is a biological marker of oxidative stress found to be increased in AMD patients [201,202]. Intravitreal injection of MDA in mice significantly enlarged the volume of laser-induced CNV lesions by inducing VEGF secretion from RPE [203]. Apurinic endonuclease 1/redox factor-1 (APE1) controls oxidative stress and regulates DNA repair [204]. An inhibitor of APE1, E3330, reduces choroidal endothelial angiogenesis [205], and intravitreal injection of E3330 decreased the development of laser-induced CNV in C57BL/6J mice [206]. All these factors have indicated a strong connection between oxidative-stress-induced RPE damage and dysfunction, resulting in VEGF overproduction and subsequent CNV formation in AMD (Figure 3).

### 6.5. In Vitro Models of RPE Oxidative Damage Further Support the Detrimental Role of Oxidative Stress

In addition to in vivo models with genetic and pharmacological approaches, RPE cell cultures are often used as supplementary tools to investigate and corroborate the role of oxidative stress in RPE damage, despite their limitations. Primary, iPS, and immortalized RPE cell lines (ARPE-19 and D407) are all widely used, and many chemical oxidants have been utilized to induce oxidative stress.

Higher cell death and apoptosis in RPE cells were found after treatment with cigarette smoke extract [207], corroborating observations in vivo. NaIO_3_ is also commonly utilized as a selective inducer of RPE cell oxidative damage and cell death in culture, similar to its use in vivo. It promotes cytosolic ROS generation and activates multiple signaling cascades such as ROS-mediated p38 and JNK activation, leading to RPE damage [208]. Hydrogen peroxide (H_2_O_2_), another oxidizing agent, is often employed to cause irreversible oxidative stress in RPE cells. In human RPE cell-D407, H_2_O_2_ induces abnormal RPE morphology, intracellular ROS generation, altered mitochondrial function, and increased apoptotic markers [209]. H_2_O_2_ also causes oxidative stress in human ARPE-19 cells with increased ROS and decreased antioxidant enzymes [210], eventually induces apoptosis, and increases cell death [211,212]. Human RPE iPS cells treated with tert-butyl hydroperoxide (t-BHP), an inducer of oxidative stress, show reduced cell viability, increases in the oxidized form of glutathione disulfide (GSSG) and a reduction in glutathione levels [213]. Hence, H_2_O_2_ and tBHP administration in RPE cell cultures demonstrates features of oxidative stress mostly via the induction of apoptosis. Primary mouse RPE cells treated with paraquat (PQ), a pesticide that can effectively induce oxidative stress and subsequent cell death, exhibit features of oxidative stress such as decreased respiration, decreased metabolic rate and impaired RPE mitochondrial function [170,214]. PQ has been utilized as a model to generate low levels of chronic oxidative stress and ROS generation [215]. In addition, the accumulation of heat shock protein (Hsp70 protein) and activation of stress-sensitive AP-1 and NF-κB were found in human ARPE-19 cells exposed to 4-hydroxynonenal (HNE)-induced oxidative stress [216], where 4-HNE instigates oxidative damage and decreases cell viability via caspase-3-dependent pathways [216]. These studies further support the detrimental role of oxidative damage in RPE health.

These RPE cell lines and oxidants were employed to probe many new regulators of the oxidative stress and defense pathways relevant to AMD. For example, activators of nuclear receptor REV-ERBα increase ARPE-19 cell viability after treatment with PQ [170]. Recently, death-associated protein like-1 (DAPL1), a candidate gene highly expressed in mature RPE cells, was associated with AMD pathogenesis [217]. *Dapl1^−/−^* RPE cells show increased oxidative stress, as evident from increased ROS production and an impaired antioxidant defense system in mice [214]. In addition, circSPECC1, a circular RNA, was recently associated with oxidative stress, irregular lipid metabolism and AMD. CircSPECC1 deficiency initiates oxidative-stress-induced ferroptosis and depolarization of RPE, as primary human fetal RPE (fRPE) and ARPE-19 cells transfected with circSPECC1-siRNA demonstrated elevation in mitochondrial ROS production [218]. The knockdown of CFH leads to reduced mitochondrial respiration, increased lipid peroxidation and ultimately to oxidative stress in hTERT-RPE1 cells, as CFH contributes to metabolic homeostasis and protects RPE cells from oxidative damage [219]. Increased complement receptors such as CR3 have also been linked with increased oxidative stress in ARPE-19 cell lines [220].

## 7. Therapeutic Roles of Antioxidants to Counter Oxidative Stress in AMD

### 7.1. Benefits of Antioxidant Intake in AMD Are Endorsed by AREDS I and II

Current treatments for wet AMD include anti-VEGF therapies, which are effective, but with limitations. For the treatment of dry AMD, anti-complement therapy was recently approved by the FDA, which has shown modest protection again progression of GA as the only treatment available for dry AMD at the time of this review. In the past few decades, two large clinical studies were undertaken: the Age-Related Eye Disease Study (AREDS) and AREDS2 sponsored by the National Eye Institute, to investigate the risk factors for AMD and the effects of antioxidants on the progression of AMD [221,222]. Dietary intake of antioxidants for slowing AMD progression was supported by findings from the AREDS and AREDS2 studies for slowing AMD progression. Additional studies support that AMD can be partially prevented or that progression can be slowed by several antioxidant molecules and antioxidant-related mechanisms to scavenge excess ROS and to protect against oxidative damage. Oral supplements of antioxidants such as vitamins C and E, zinc, lutein, zeaxanthin, and beta carotene decrease the likelihood of progression of AMD [47], as supported by AREDS studies.

The original AREDS study included 3640 participants and indicated that both zinc (80 mg, zinc oxide) and antioxidants (500 mg vitamin C, 400 IU vitamin E, and 15 mg beta carotene) or the combination of both significantly minimized the odds of progressing to advanced AMD [221]. Zinc, a key AREDS ingredient, is a crucial component of many enzymes in maintaining retinal health and metabolism [223,224]. In AREDS2, a formulation was improved by replacing beta-carotene with lutein and zeaxanthin and by reducing zinc, which can further decrease the risk of AMD progression [221,222]. Lutein and zeaxanthin are enriched in the macula region of the retina and are known components of macular pigment. They play important roles in minimizing the oxidative damage and in decreasing the risk of AMD as ingredients of the AREDS2 formula [222,225,226].

The addition of omega 3 fatty acids to the AREDS2 formula, however, did not show significant protection, despite strong protective effects in experimental studies and in clinical trials of cardiovascular diseases [227]. A clinical study reported that the supplementation of omega-3 fatty acids when combined with anti-VEGF is linked with decreased levels of vitreal VEGF-A in wet AMD patients [228].

Although studies have supported the efficacy and beneficial impacts of these antioxidants such as vitamins (A, C, E), zinc, lutein and zeaxanthin, their formulations are not universally endorsed, as they have their own pros and cons, which should be carefully considered, particularly in terms of potential interactions with patient genotypes [229]. Overall, these antioxidant supplements have shown great promise to protect against AMD progression (Figure 3).

### 7.2. Other Antioxidants in Protecting against AMD: Support from Additional Studies

A robust correlation has been established between oxidative stress and inflammation in the pathophysiology of AMD. Oxidative stress plays a major role in AMD pathology by different routes such as dysfunctional autophagy machinery, apoptosis, DNA damage, defects in antioxidant enzymes, and deficiency in antioxidants, which together increase the RPE oxidative damage and worsen AMD features [230]. Dietary molecules are a crucial source of antioxidants for regulating oxidative stress and hence are widely investigated for their ability to protect against RPE damage and CNV.

In addition to AREDS antioxidant ingredients, polyphenols, a group of plant-derived compounds with antioxidant capacity, are also crucial in scavenging ROS, reducing inflammation, and improving ocular blood flow. Polyphenols diminish RPE death, opacification of the lens, and BRB breakdown [231]. Resveratrol, an antioxidant and polyphenol phytoalexin rich in many plant products and fruits [232], prevents excessive VEGF production and secretion in ARPE-19 cells subjected to oxidative stress [233] and in human retinal pigment epithelial cultures [234], and it protects against blue-light-induced ARPE-19 cell death [235] and laser-induced CNV [236,237]. In a mouse model of CNV, a strong correlation of omega-3 long-chain-polyunsaturated fatty acid in decreasing CNV lesions has been found [238], in part acting through adiponectin [227] and cytochrome P450 enzyme metabolites [239,240]. Moreover, a combined omega-3/resveratrol supplement showed significant protection against CNV compared to each single supplement alone [241]. Cerium oxide nanoparticles (CeO2-NPs) are considered a promising antioxidant nanomaterial for AMD [242], which can reduce the detrimental effects of H_2_O_2_ -induced oxidative stress in human umbilical vein endothelial cells (HUVECs) [243]. CeO2-NPs prevent retinal neovascularization in vitro and in *Vldlr^−/−^* mice [244,245] and protect ARPE-19 cells [246]. Antioxidant enzymes such as SOD1 and SOD2 play a pivotal role in scavenging ROS generation and are reported to be beneficial in protecting against AMD progression [247]. Glutathione (GSH) is an antioxidant, the levels of which correlate with macular pigment optical density [248,249]. GSH is protective against oxidative damage, and GSH depletion promotes ferroptosis, autophagic mechanisms and cellular senescence [250]. GSH protects RPE cells against oxidative stress [250,251]. N-acetyl-cysteine (NAC), an antioxidant precursor of glutathione, also inhibits oxidative stress, protects RPE, decreases NF-κB activation and suppresses the development of CNV in C57BL/6 mice [251,252]. The beneficial role of these antioxidant molecules and enzymes can be combined with the AREDS antioxidants as described above to reduce the progression of RPE loss and CNV [221,253] (Figure 3). A summary of these selected antioxidants in clinical and experimental studies of AMD can be found in Table 1.

## 8. Conclusions

The retina is one of the most oxygen-sensitive tissues and is thus highly susceptible to oxidative stress. With age, multiple cell types in the eye accumulate oxidative damage that contributes in part to the onset and progression of age-related eye diseases with visual impairment, including glaucoma, cataracts, and AMD. In this review, we have highlighted evidence for the pathogenic role of oxidative stress in clinical and experimental AMD and the potential of antioxidants as viable options to counter oxidative damage to protect RPE and to prevent CNV. In large clinical studies such as AREDS and AREDS2, antioxidants have been proven to be associated with reduced risk of progression to advanced AMD. Experimental studies on oxidative regulatory pathways and antioxidant treatment were also discussed, with supporting findings in both dry and wet AMD settings and models. Antioxidants might thus be effective in AMD intervention by delaying or reducing cellular oxidative damage in the RPE and the retina. Additional studies will be required to elucidate the interaction between oxidative stress and other pathogenic processes in AMD, including innate immunity, chronic inflammation, and lipid metabolism. Identification of novel antioxidants and phytochemicals is warranted as potential treatments, with further work needed on their detailed mechanisms of action, bioavailability, and pharmacokinetics.

## Figures and Tables

**Table 1:** Summary of selected clinical and experimental studies on antioxidants in AMD.

Antioxidants	Chemical Nature	Dietary Sources	Functions	Effects in AMD	References
**Clinical Studies**					
Vitamins C and E	Vitamin C as ascorbic acid; Vitamins E as tocopherol and tocotrienol.	Vitamin C: citrus fruits, berries, and red pepper Vitamin E: nuts, seeds, greens, whole grains.	Vitamin C reduces oxidative stress and promotes immune protection. Vitamin E maintains retinal structure and function and scavenges peroxyl radicals.	Low intake was associated with neovascular AMD. Protective against AMD progression as a component of AREDS and AREDS2 formula.	[221,222,253,254,255,256]
Vitamin A, β-carotene	Pure form of vitamin A as retinol; β-carotene as precursor of vitamin A.	Green leafy vegetables, fruits, dairy products, fish, and eggs.	Vitamin A aids formation of photopigment to support vision, also quenches free radicals.	β-carotene is protective against AMD progression as a component of AREDS formula.	[221,222,229,256,257,258]
Zinc	Essential trace mineral, and micronutrients	Oysters, meat, seafood, fish, poultry, cereals, and grains.	Functions as a co-factor of antioxidant enzymes and protects against oxidative damage.	Significantly reduced the risk of developing advanced AMD, as a component of AREDS and AREDS2 formula.	[221,222,259]
Lutein and zeaxanthin.	Xanthophyll family of carotenoids	Green leafy vegetables, egg yolks, yellow-orange fruits.	Functions as components of macular pigment, filters blue light and quenches single oxygen and free radicals.	Reduces the risk of AMD progression. Replaces β-carotene in AREDS2 formula to reduce cancer risk.	[222,260,261,262,263]
ω-3 long-chain polyunsaturated fatty acid (LCPUFA)	Mainly as eicosapentaenoic (EPA, 20:5 ω-3), and docosahexaenoic (DHA, 22:6 ω-3) acids	Nuts and seeds, plant oils, nuts, fish, and seafood.	Supports photoreceptor membrane structure, reduces inflammation, and limits oxidative stress.	Lower intake increases the risk of neovascular AMD, and higher intake was associated with decreased risk of AMD in prospective studies. Yet addition to AREDS2 did not further reduce risk of AMD progression.	[222,261,264,265,266,267,268]
Resveratrol	Polyphenols	Grape juices, wines, berries, and peanuts.	Protects against oxidative stress, anti-inflammatory.	Addition to AREDS EU formula did not have significant effects in wet AMD. Another clinical trial result pending.	[269,270]
Cu/Zn superoxide dismutase (SOD1)	Antioxidant enzymes	Supplementing copper may increase SOD levels in endogenous synthesis.	Scavenges superoxide anion and protects against oxidative stress.	Decreased in erythrocytes and serum of AMD patients in a few studies yet increased in other studies reflecting potential compensation. Increased SOD protein observed in AMD doner eyes.	[125,128,129,130,131]
MnSOD (SOD2)	Antioxidant enzymes	Manganese is required for endogenous synthesis.	A mitochondrial enzyme detoxifies free radicals from mitochondrial respiration.	Lowest expression was observed in wet and dry form of AMD, with genetic correlation. Increased protein levels in AMD donor eyes.	[125,271]
**Experimental Studies**					
ω-3 long-chain polyunsaturated fatty acid (LCPUFA)	Mainly as eicosapentaenoic (EPA, 20:5 ω-3), and docosahexaenoic (DHA, 22:6 ω-3) acids	Nuts and seeds, plant oils, nuts, fish, and seafood.	Supports photoreceptor membrane structure, reduces inflammation, and limits oxidative stress.	Inhibits laser-induced CNV in mouse models.	[227,238,239,240,241]
Resveratrol	Polyphenols	Grape juices, wines, berries, peanuts.	Protects against oxidative stress, anti-inflammatory.	Suppresses laser-induced CNV. Prevents excessive VEGF production.	[233,234,235,236,237]
SOD1 and SOD2	Antioxidant enzymes	Endogenous synthesis. Requires trace minerals Cu, Zn, Mn.	Scavenges superoxide anion in cytosol and in mitochondria and protects against oxidative stress.	SOD1 knockout and SOD2 down lead to AMD-like features in mice with RPE degeneration. A small fraction of SOD1 knockout mice develop CNV.	[142,143,144]
Glutathione (GSH)	Tripeptide consisting of three amino acids: glutamic acid, cysteine, and glycine	Produced endogenously in the liver. Adding sulfur- and selenium-rich food helps.	Scavenges electrophilic and oxidant species to reduced oxidative stress.	Protects RPE cells from oxidative damage in ARPE19 and primary RPE isolated from AMD donors.	[248,249,250,251]
N-acetyl-cysteine (NAC)	A precursor of L-cysteine, aids synthesis of glutathione	Seafood, chicken, turkey, fish, protein-rich foods.	Antioxidant and cytoprotectant.	Reduces oxidative damage in RPE and suppresses laser-induced CNV in mice.	[251,252]
Cerium oxide nanoparticles (CeO2-NPs, nanoceria)	Nanomaterial	Not applicable. Synthetic material.	Inorganic antioxidants mimicking SOD and catalase.	Prevents retinal neovascularization in vitro and in *Vldlr^−/−^* mice. Protects RPE from oxidative damage.	[242,243,244,245,246]
